# Supplementary material for: Phytoceramides from the Marine Sponge Monanchora clathrata: Structural Analysis and Cytoprotective Effects
Source: Biomolecules. 2023 Apr 14;13(4):677. doi: 10.3390/biom13040677 (PMC10136155; doi:10.3390/biom13040677)
Supplement: Supplementary file 1 [file biomolecules-13-00677-s001.zip › biomolecules-2281782-supplementary.pdf]

Supplementary Materials for the Article

Phytoceramides from the marine sponge *Monanchora clathrata*: Structural Analysis and Cytoprotective Effects

Elena A. Santalova *, Alexandra S. Kuzmich, Ekaterina A. Chingizova, Ekaterina S. Menchinskaya, Evgeny A. Pislyagin and Pavel S. Dmitrenok*

G.B. Elyakov Pacific Institute of Bioorganic Chemistry, Far Eastern Branch of the Russian Academy of Sciences, Pr. 100 let Vladivostoku 159, 690022 Vladivostok, Russia

Content

Figure S1. ^{1}H-NMR spectrum (**a**–**c**) of total ceramide (700 MHz, C_5D_5N)……………………………………………………………………………....2–4
Figure S2. ^{13}C-NMR spectrum of total ceramide (125 MHz, C_5D_5N)…………………………………………………………………………………..5
Figure S3. HR-ESI-MS analyses (negative ion mode) of total ceramide……………………………………………………………………………..6
Figure S4. (–)ESI-MS/MS spectrum of ion at *m*/*z* 640.59 ([M – H]$^-$)………………………………………………………………………………....7
Figure S5. (–)ESI-MS/MS spectrum of ion at *m*/*z* 654.60 ([M – H]$^-$), illustrated with fragmentation pattern of compound **3b**……………………..8
Figure S6. (–)ESI-MS/MS spectrum of ion at *m*/*z* 668.62 ([M – H]$^-$), illustrated with fragmentation patterns of compounds **2e**, **3c**, and **6b**……………..9, 10
Figure S7. (–)ESI-MS/MS spectrum of ion at *m*/*z* 682.63 ([M – H]$^-$), illustrated with fragmentation patterns of compounds **2f**, **3e**, and **6c**……………11, 12
Figure S8. (–)ESI-MS/MS spectrum of ion at *m*/*z* 696.65 ([M – H]$^-$), illustrated with fragmentation patterns of compounds **3f** and **6e**………………13, 14
Figure S9. (–)ESI-MS/MS spectrum of ion at *m*/*z* 710.665 ([M – H]$^-$), illustrated with fragmentation patterns of compounds **3g** and **6f**……………15, 16
Figure S10. (–)ESI-MS/MS spectrum of ion at *m*/*z* 724.68 ([M – H]$^-$), illustrated with fragmentation patterns of compounds **5g** and **6g**……………..17
Figure S11. Cytotoxicities of total ceramide against (**a**) MDA-MB-231 and (**b**) HL-60 cell lines…………………………………………………..18
Figure S12. Cytotoxicities of crambescidin 359 against (**a**) MDA-MB-231and (**b**) HL-60 cell lines……………………………………………..18
Figure S13. Protective effects of crambescidin 359 against neurotoxicity of paraquat (PQ): (**a**) Neuro-2a cells (24 h pre-incubation) and (**b**) SH-SY5Y cells (48 h pre-incubation)……………………………………………………………………………………………..19

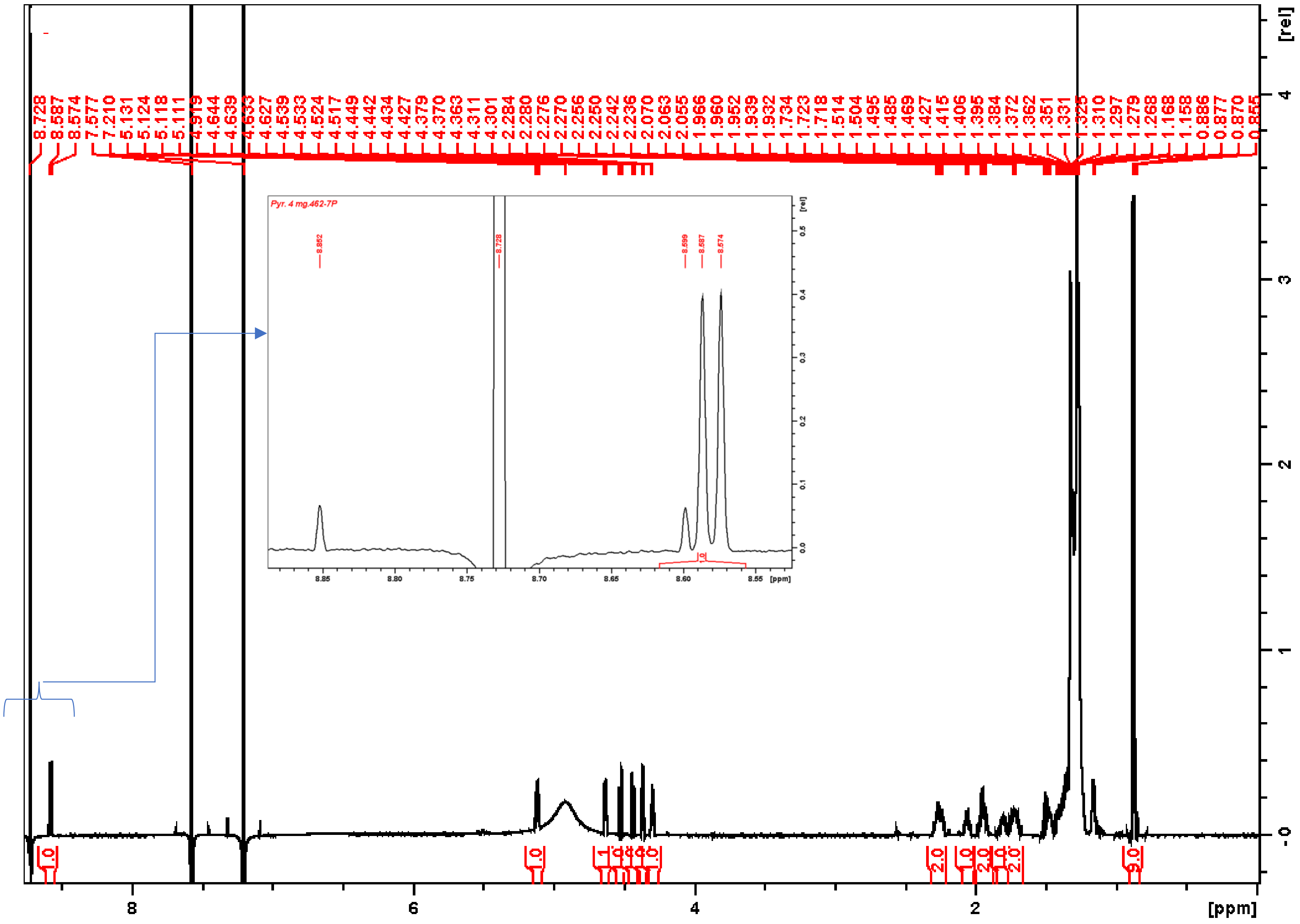

(a)

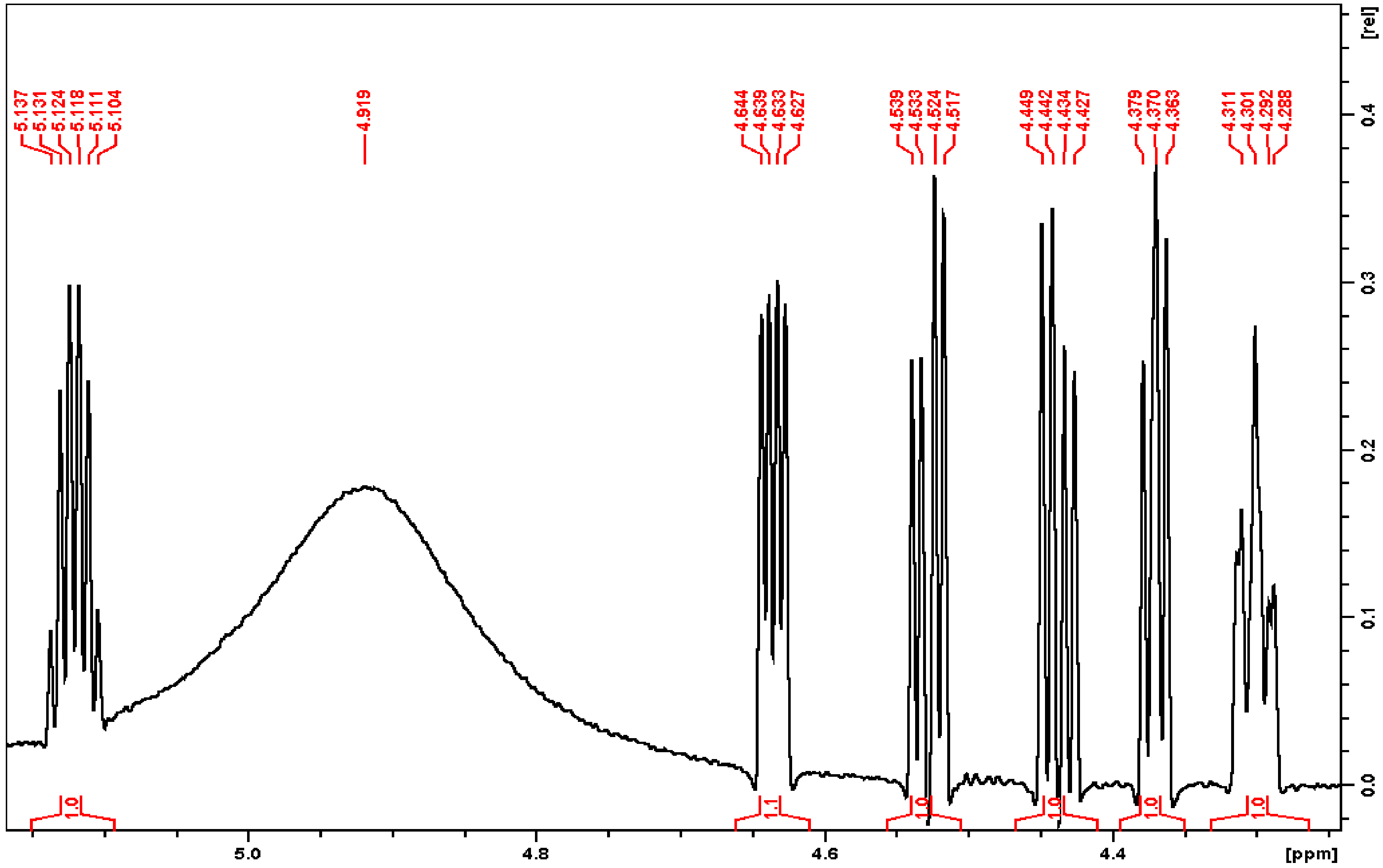

5.137
5.131
5.124
5.118
5.111
5.104
4.919
4.644
4.639
4.633
4.627
4.539
4.533
4.524
4.517
4.449
4.442
4.434
4.427
4.379
4.370
4.363
4.311
4.301
4.292
4.288
1.0
1.1
1.0
1.0
1.0
1.0
[rel]
0.4
0.3
0.2
0.1
0.0
5.0
4.8
4.6
4.4
[ppm]

(b)

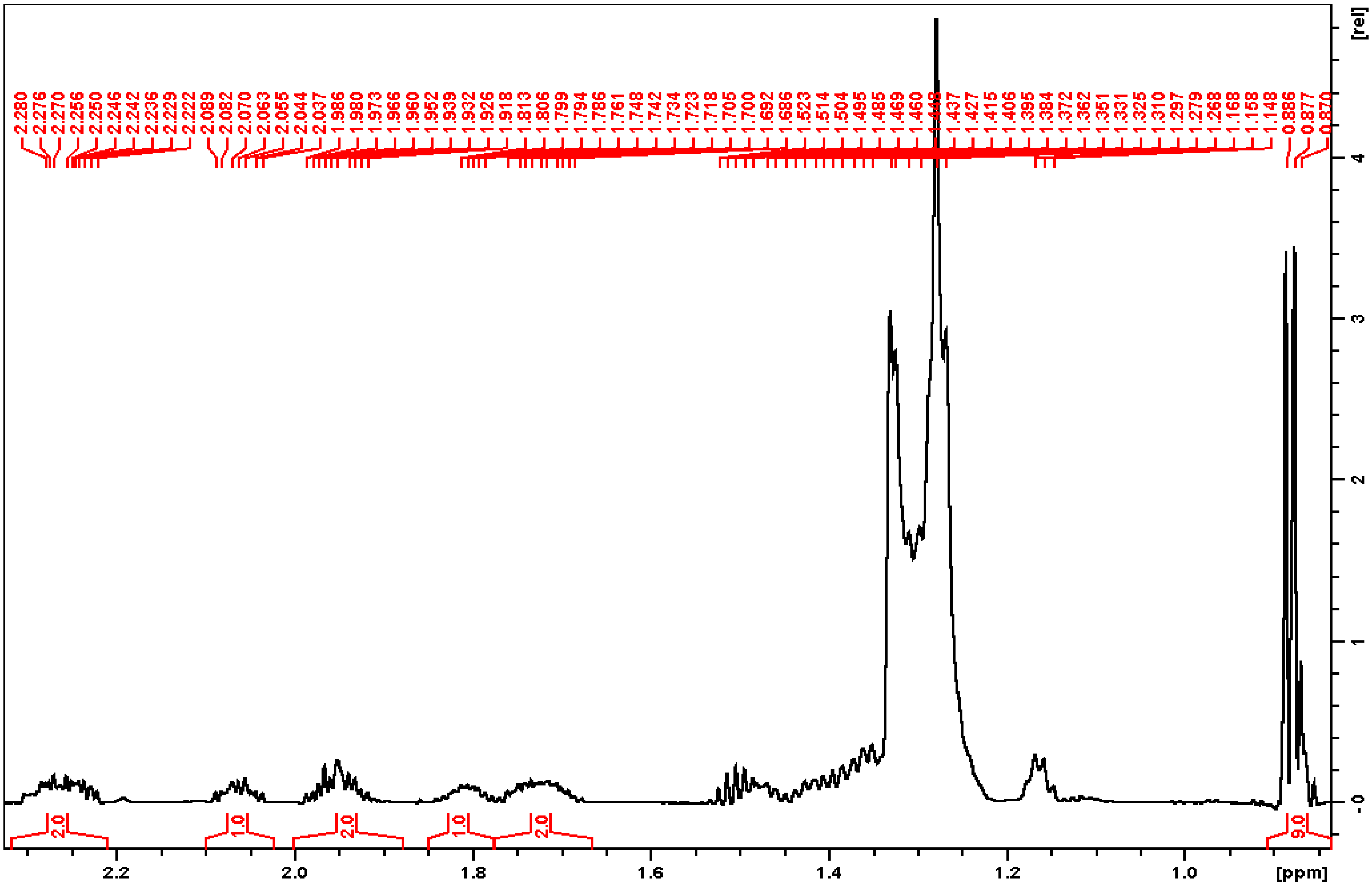

(c)

Figure S1. ^{1}H-NMR spectrum (**a–c**) of total ceramide (700 MHz, C_5D_5N).

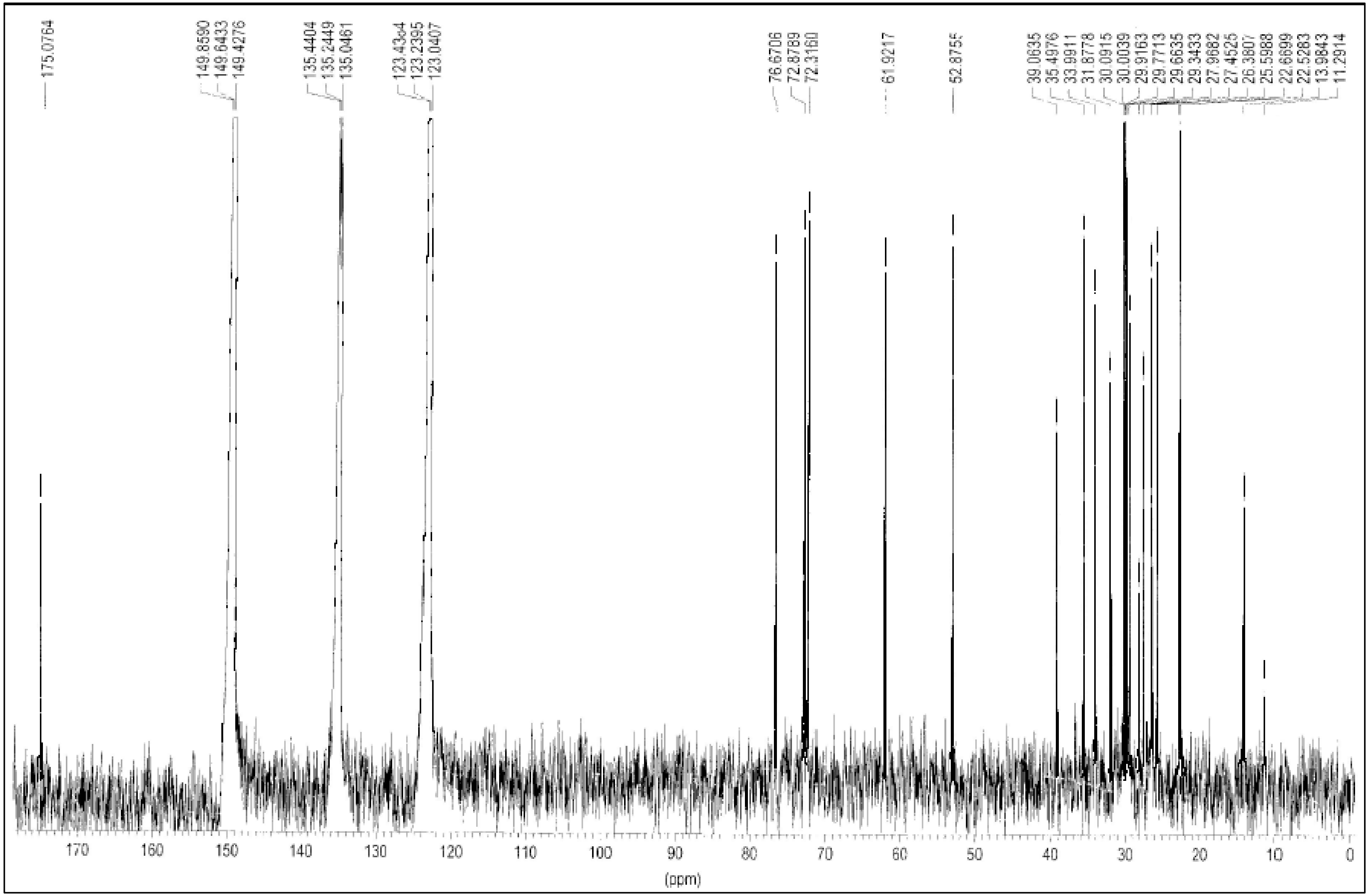

Figure S2. ^{13}C-NMR spectrum of total ceramide (125 MHz, C_5D_5N).

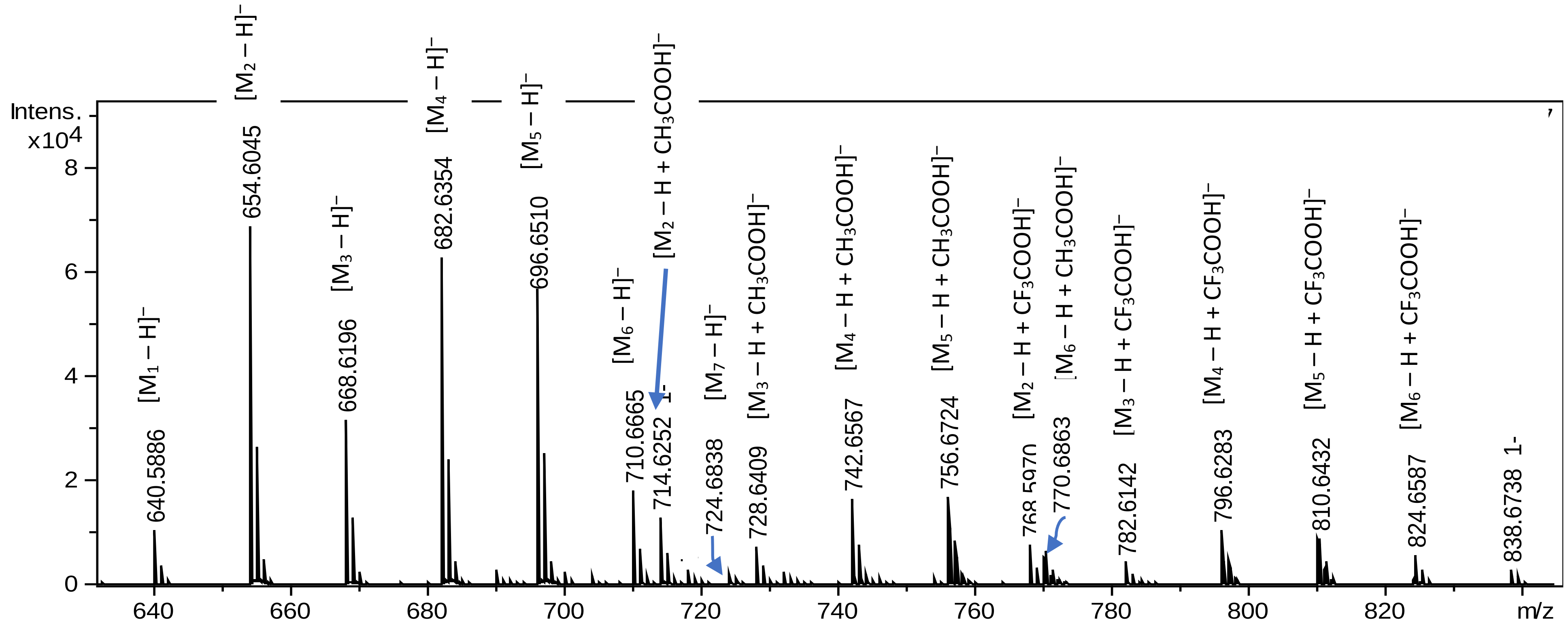

Figure S3. HR-ESI-MS analyses (negative ion mode) of total ceramide.

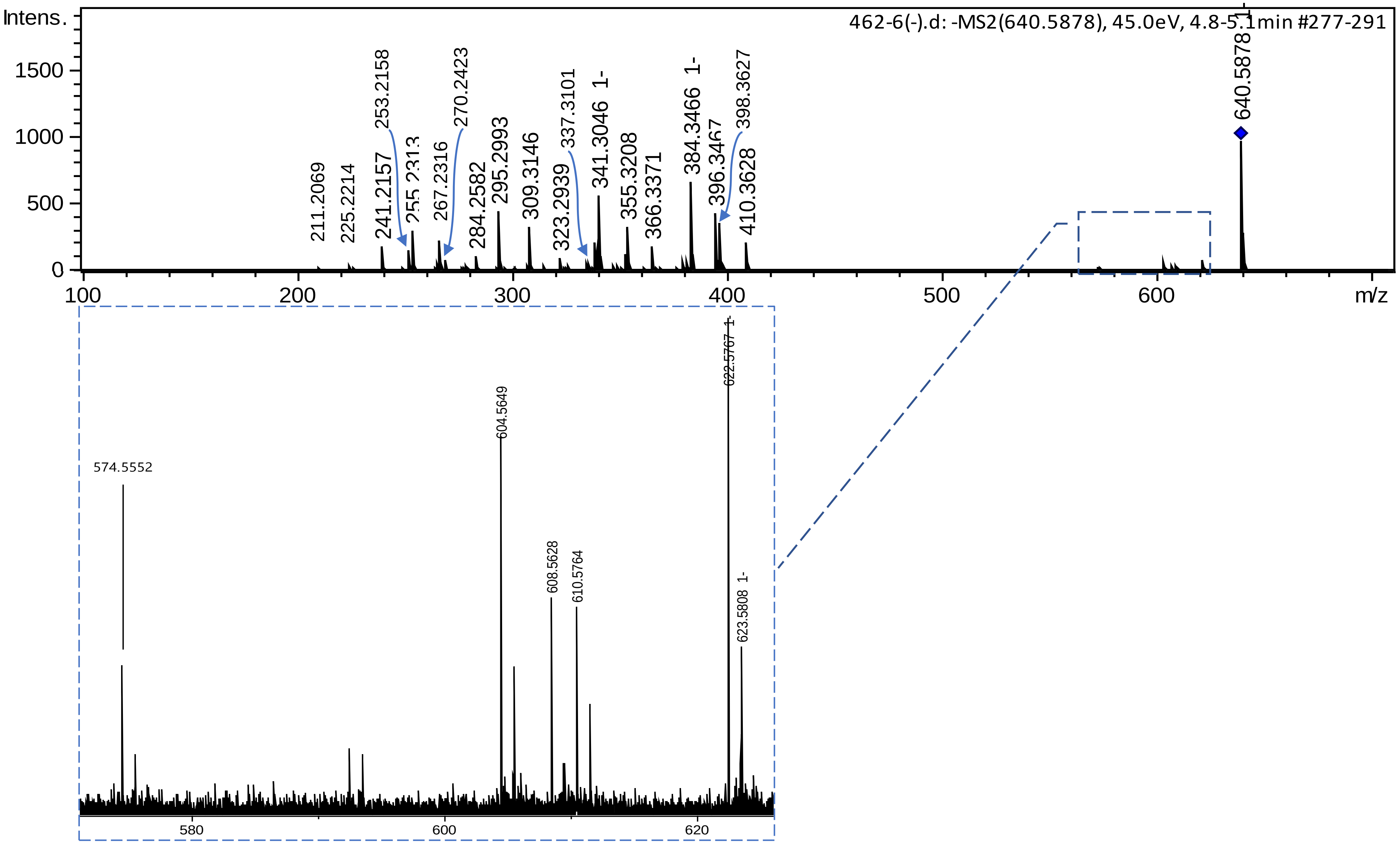

Figure S4. (–)ESI-MS/MS spectrum of ion at *m*/*z* 640.59 ([M – H]⁻).

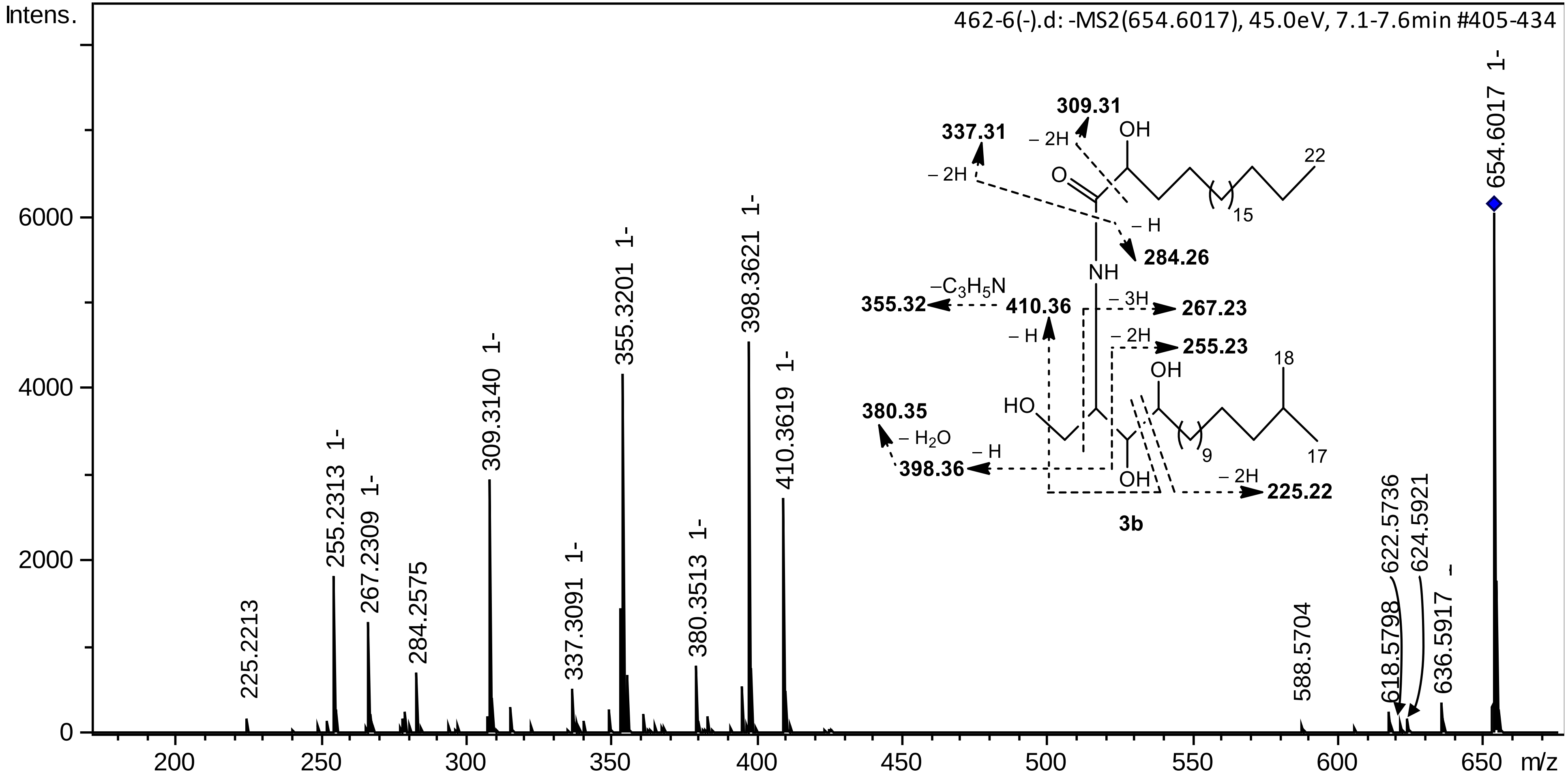

Figure S5. (–)ESI-MS/MS spectrum of ion at *m*/*z* 654.60 ([M – H]⁻), illustrated with fragmentation pattern of compound **3b**.

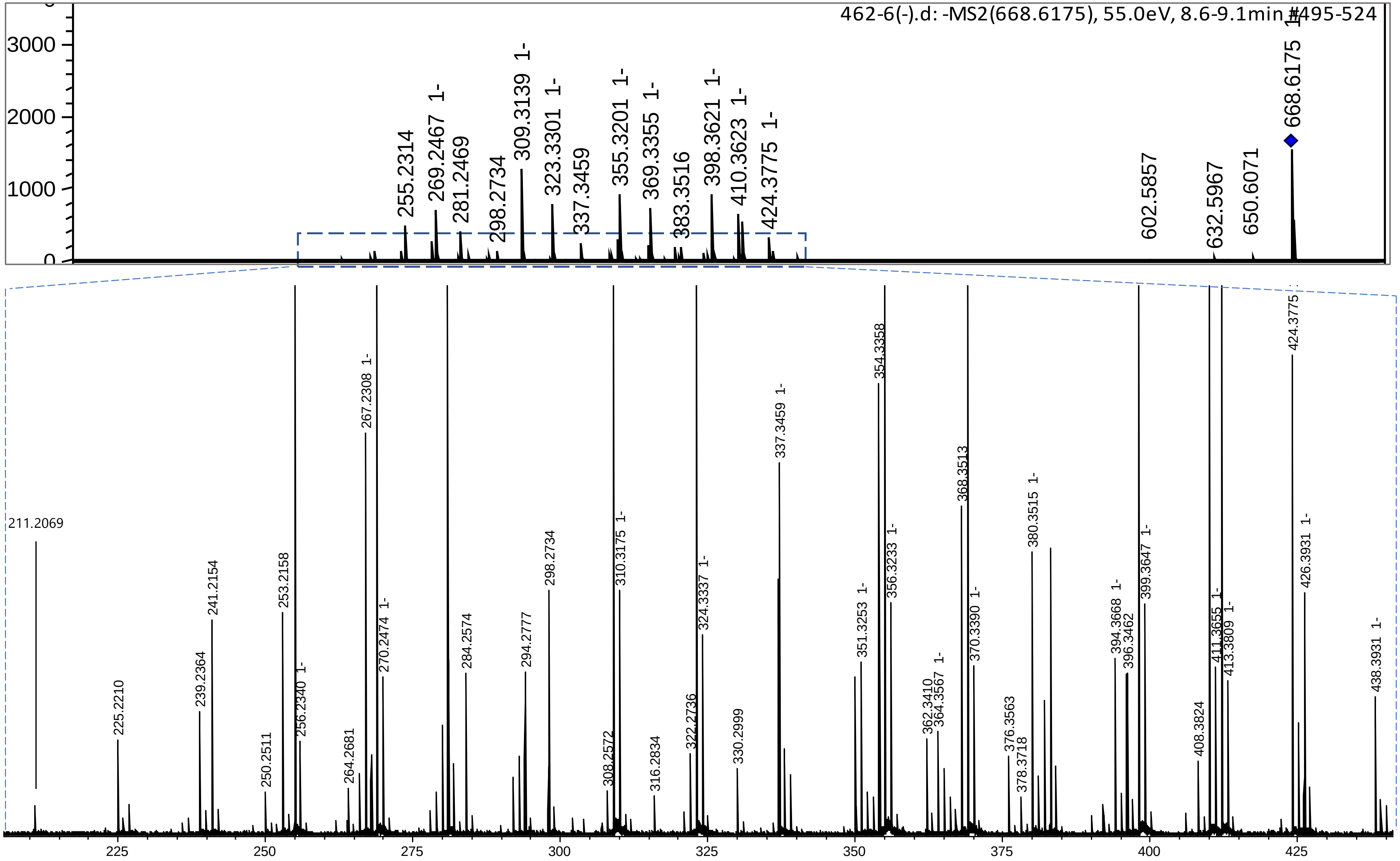

462-6(-).d: -MS2(668.6175), 55.0eV, 8.6-9.1min #495-524
3000
2000
1000
0
255.2314
269.2467 1-
281.2469
298.2734
309.3139 1-
323.3301 1-
337.3459
355.3201 1-
369.3355 1-
383.3516
398.3621 1-
410.3623 1-
424.3775 1-
602.5857
632.5967
650.6071
668.6175 1-
211.2069
225.2210
239.2364
241.2154
250.2511
253.2158
256.2340 1-
264.2681
267.2308 1-
270.2474 1-
284.2574
294.2777
298.2734
308.2572
310.3175 1-
316.2834
322.2736
324.3337 1-
330.2999
337.3459 1-
351.3253 1-
354.3358
356.3233 1-
362.3410
364.3567 1-
368.3513
370.3390 1-
376.3563
378.3718
380.3515 1-
394.3668 1-
396.3462
399.3647 1-
408.3824
411.3655 1-
413.3809 1-
424.3775
426.3931 1-
438.3931 1-
225
250
275
300
325
350
375
400
425

(a)

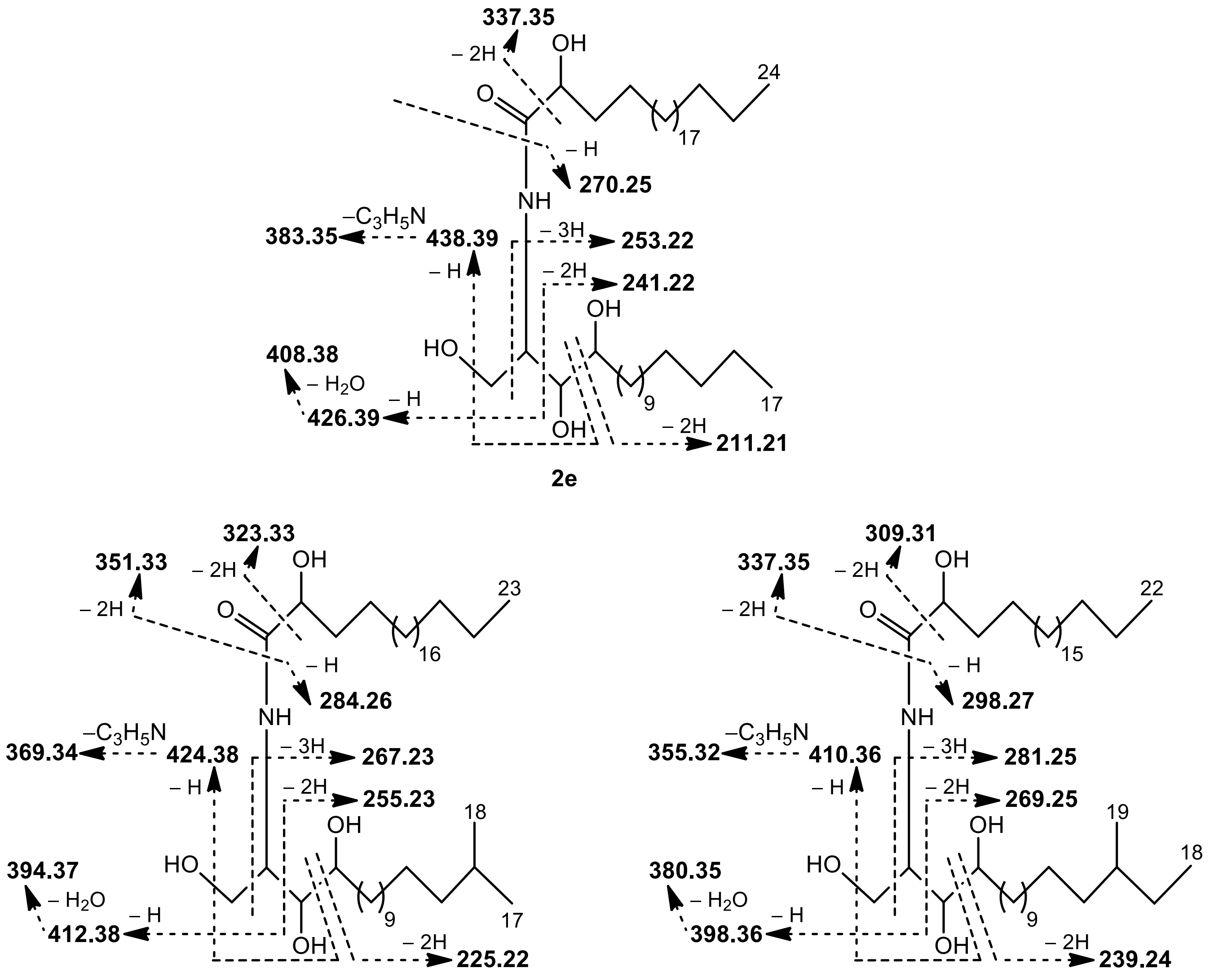

3c **6b**

(**b**)

Figure S6. (–)ESI-MS/MS spectrum of ion at *m*/*z* 668.62 ([M – H]⁻), illustrated with fragmentation patterns of compounds **2e**, **3c**, and **6b**.

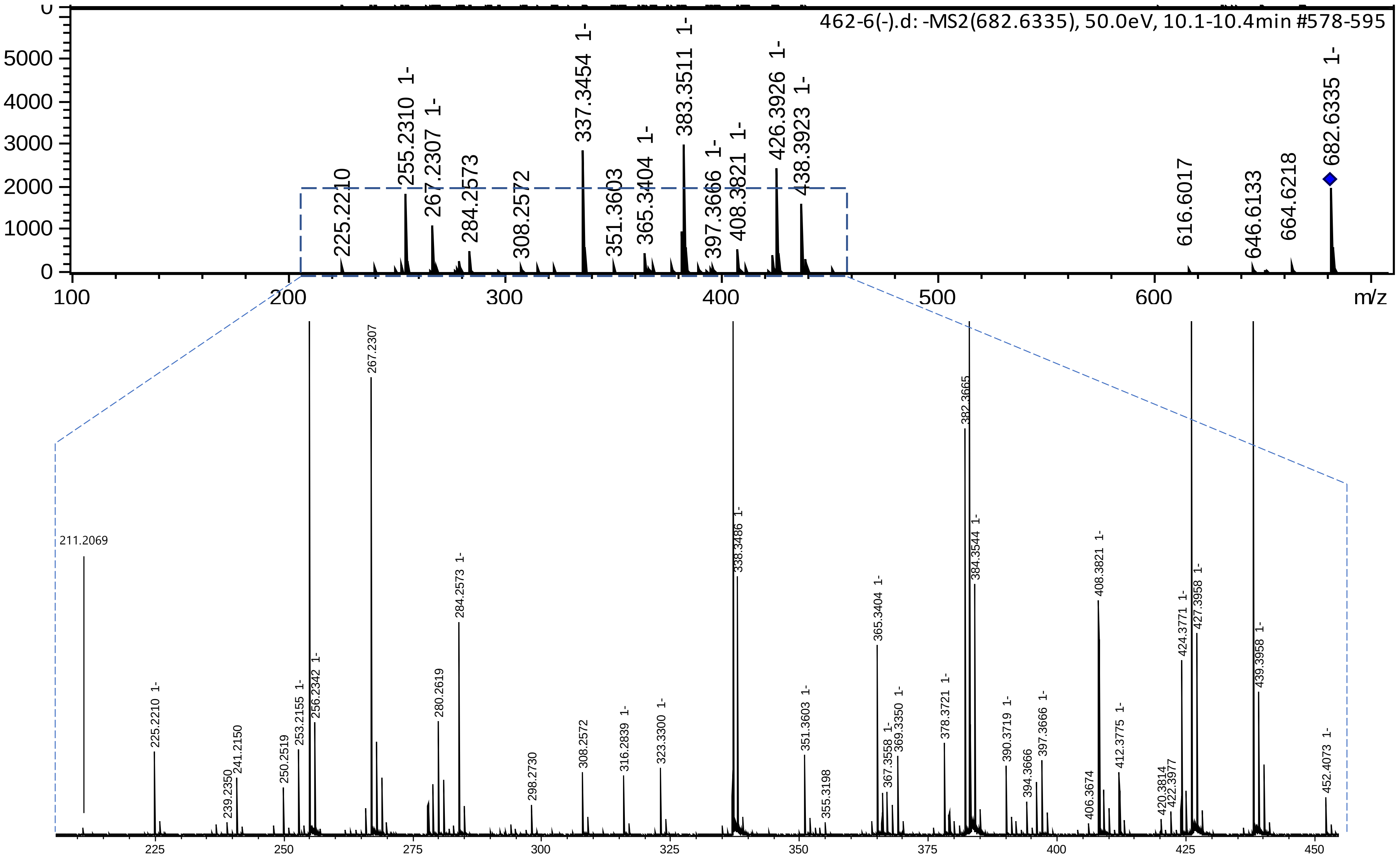
462-6(-).d: -MS2(682.6335), 50.0eV, 10.1-10.4min #578-595
5000
4000
3000
2000
1000
0
100
200
300
400
500
600
m/z
225.2210
255.2310 1-
267.2307 1-
284.2573
308.2572
337.3454 1-
351.3603
365.3404 1-
383.3511 1-
397.3666 1-
408.3821 1-
426.3926 1-
438.3923 1-
616.6017
646.6133
664.6218
682.6335 1-
211.2069
225.2210 1-
239.2350
241.2150
250.2519
253.2155 1-
256.2342 1-
267.2307
280.2619
284.2573 1-
298.2730
308.2572
316.2839 1-
323.3300 1-
338.3486 1-
351.3603 1-
355.3198
365.3404 1-
367.3558 1-
369.3350 1-
378.3721 1-
382.3665
384.3544 1-
390.3719 1-
394.3666
397.3666 1-
406.3674
408.3821 1-
412.3775 1-
420.3814
422.3977
424.3771 1-
427.3958 1-
439.3958 1-
452.4073 1-
225
250
275
300
325
350
375
400
425
450

(**a**)

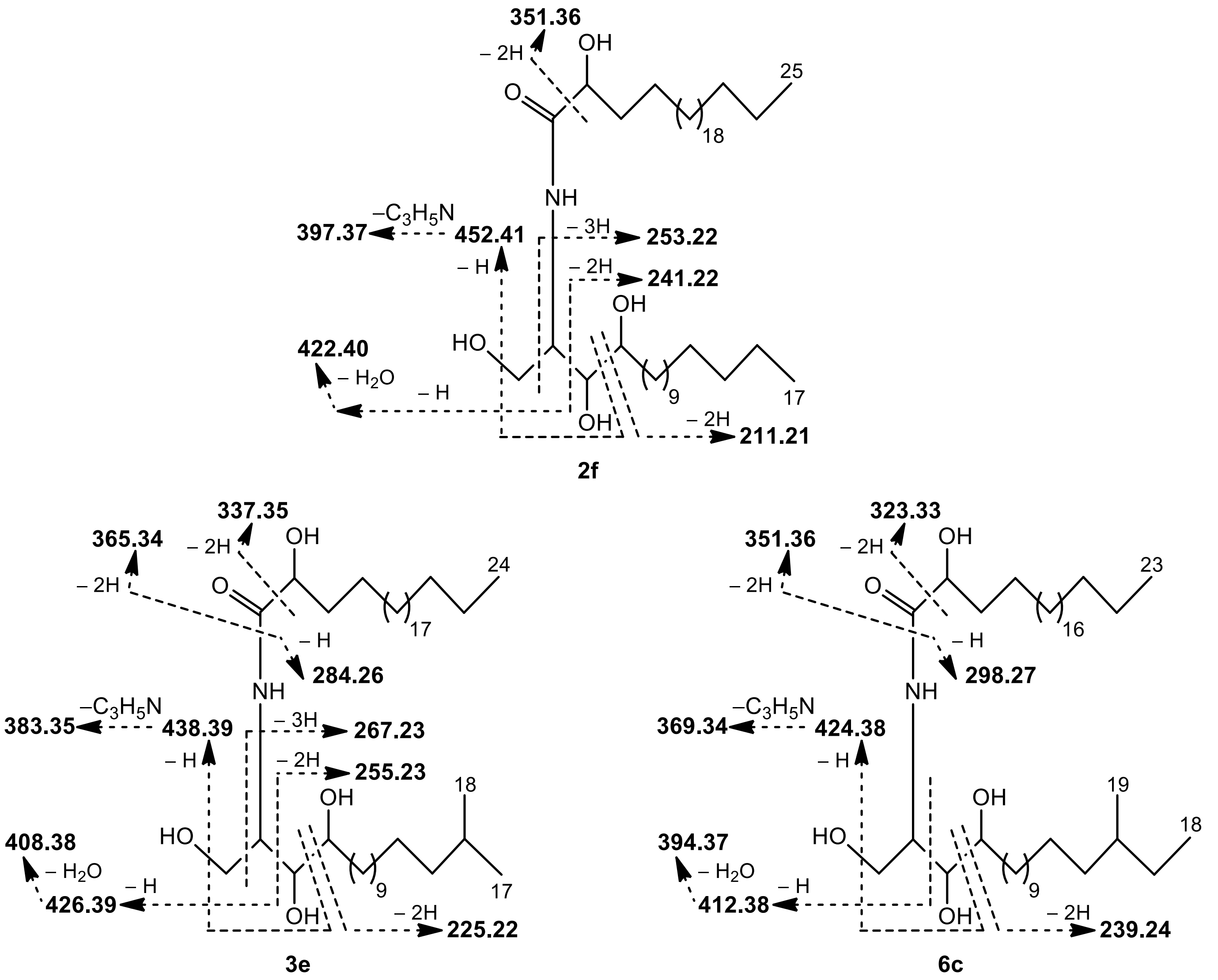

(**b**)

Figure S7. (–)ESI-MS/MS spectrum of ion at *m*/*z* 682.63 ([M – H]⁻), illustrated with fragmentation patterns of compounds **2f**, **3e**, and **6c**.

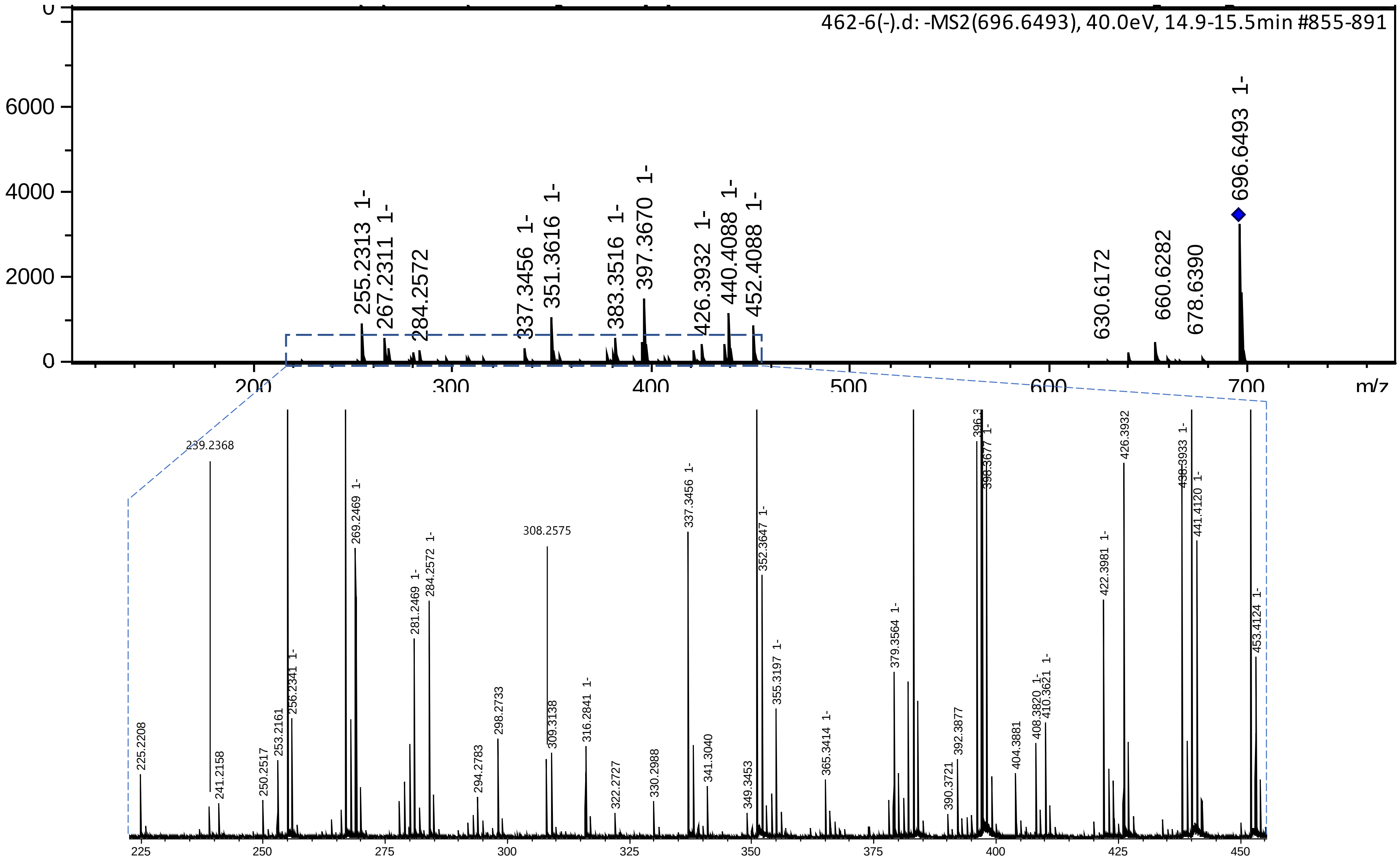

462-6(-).d: -MS2(696.6493), 40.0eV, 14.9-15.5min #855-891
6000
4000
2000
0
200
300
400
500
600
700
m/z
255.2313 1-
267.2311 1-
284.2572
337.3456 1-
351.3616 1-
383.3516 1-
397.3670 1-
426.3932 1-
440.4088 1-
452.4088 1-
630.6172
660.6282
678.6390
696.6493 1-
225.2208
239.2368
241.2158
250.2517
253.2161
256.2341 1-
269.2469 1-
281.2469 1-
284.2572 1-
294.2783
298.2733
308.2575
309.3138
316.2841 1-
322.2727
330.2988
337.3456 1-
341.3040
349.3453
352.3647 1-
355.3197 1-
365.3414 1-
379.3564 1-
390.3721
392.3877
398.3677 1-
404.3881
408.3820 1-
410.3621 1-
422.3981 1-
426.3932
438.3933 1-
441.4120 1-
453.4124 1-
225
250
275
300
325
350
375
400
425
450

(a)

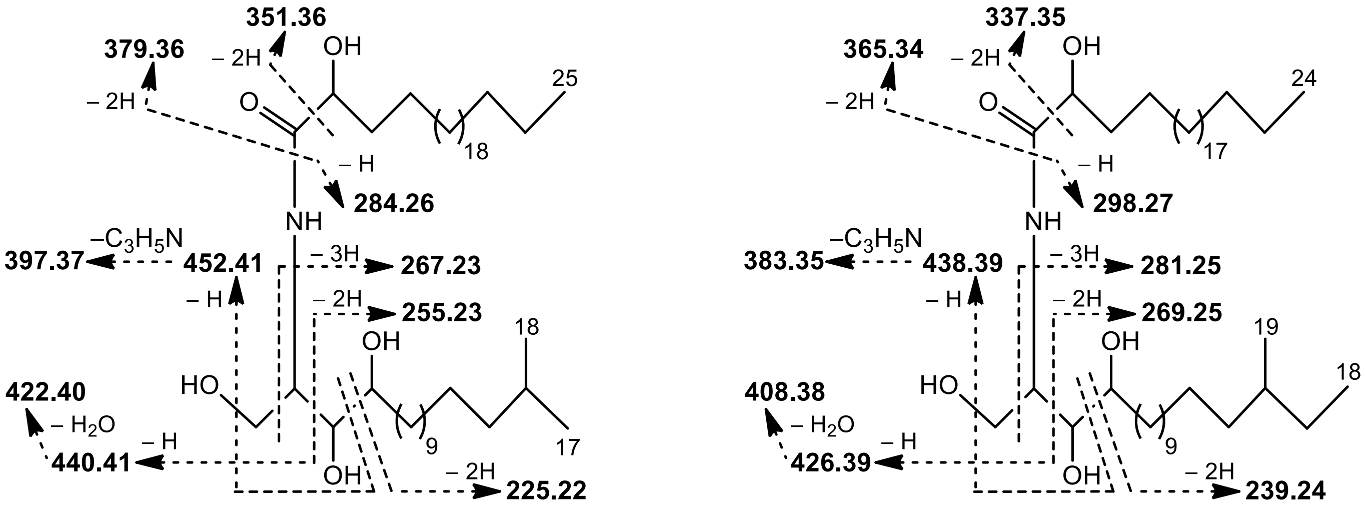

3f **6e**

(**b**)

Figure S8. (–)ESI-MS/MS spectrum of ion at *m*/*z* 696.65 ([M – H]⁻), illustrated with fragmentation patterns of compounds **3f** and **6e**.

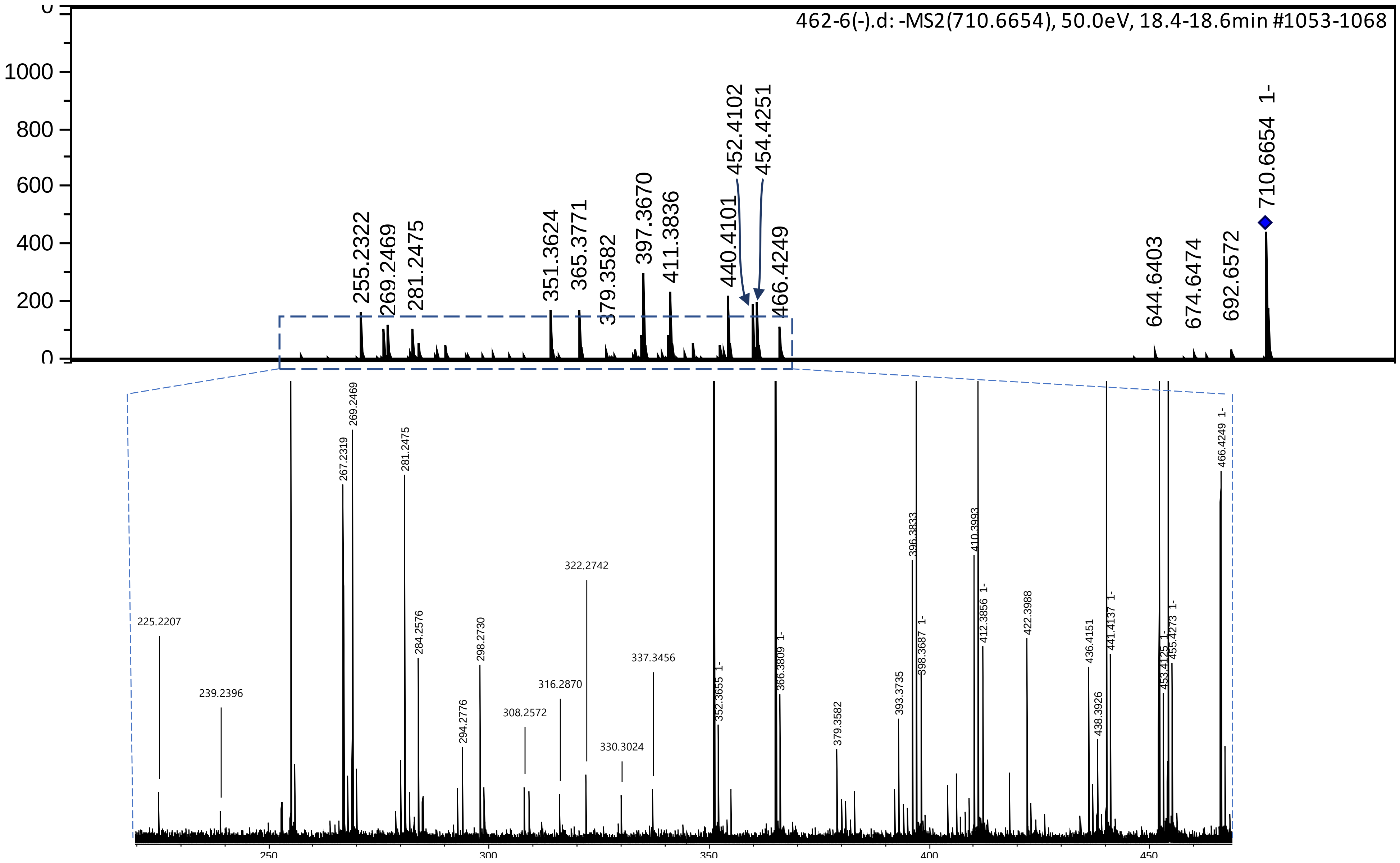
462-6(-).d: -MS2(710.6654), 50.0eV, 18.4-18.6min #1053-1068
255.2322
269.2469
281.2475
351.3624
365.3771
379.3582
397.3670
411.3836
440.4101
452.4102
454.4251
466.4249
644.6403
674.6474
692.6572
710.6654 1-
225.2207
239.2396
267.2319
269.2469
281.2475
284.2576
294.2776
298.2730
308.2572
316.2870
322.2742
330.3024
337.3456
352.3655 1-
366.3809 1-
379.3582
393.3735
396.3833
398.3687 1-
410.3993
412.3856 1-
422.3988
436.4151
438.3926
441.4137 1-
453.4125 1-
455.4273 1-
466.4249 1-

(**a**)

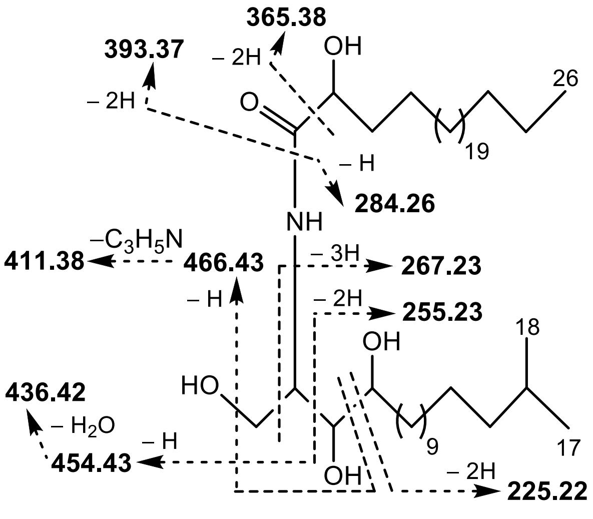

3g

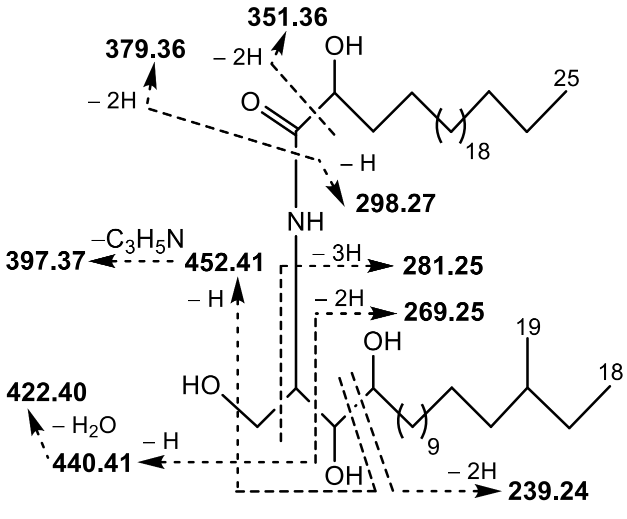

6f

(**b**)

Figure S9. (–)ESI-MS/MS spectrum of ion at *m*/*z* 710.665 ([M – H]⁻), illustrated with fragmentation patterns of compounds **3g** and **6f**.

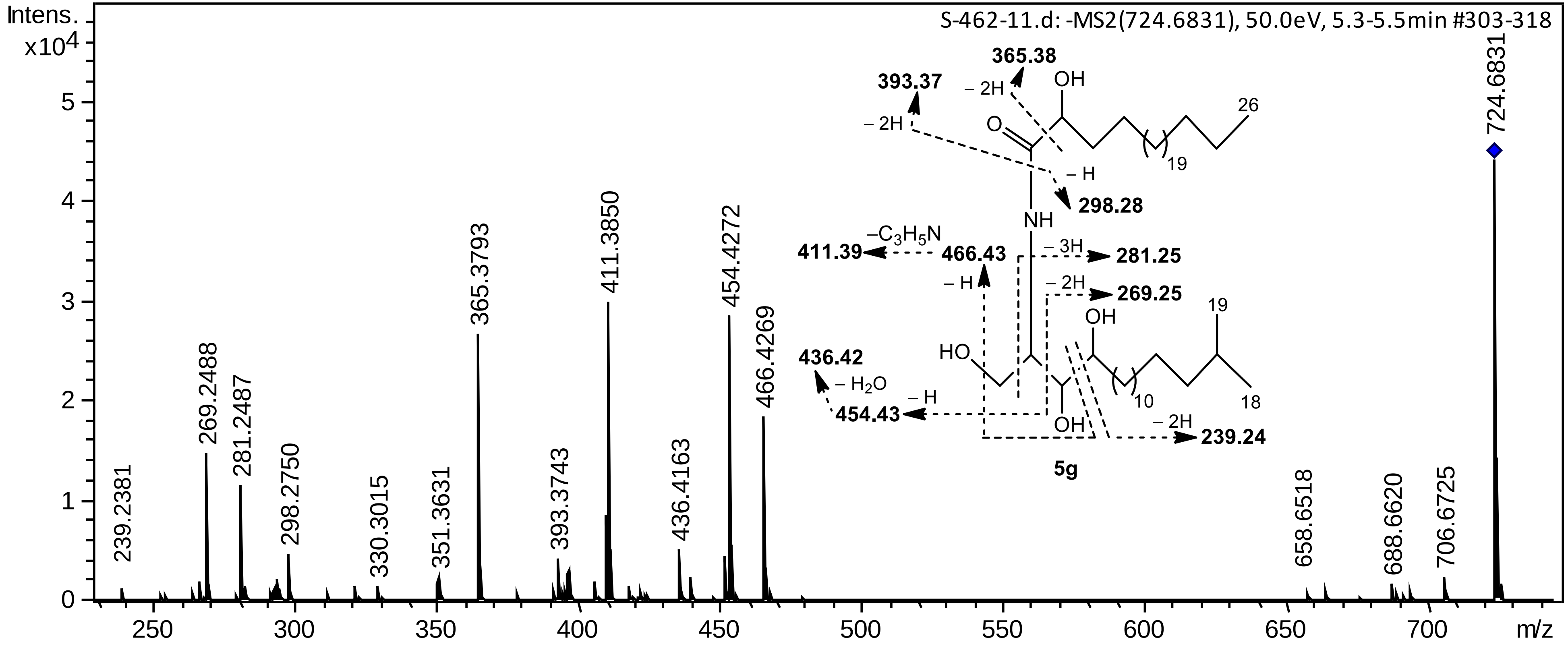

Figure S10. (–)ESI-MS/MS spectrum of ion at *m*/*z* 724.68 ([M – H]⁻), illustrated with fragmentation patterns of compound **5g**.

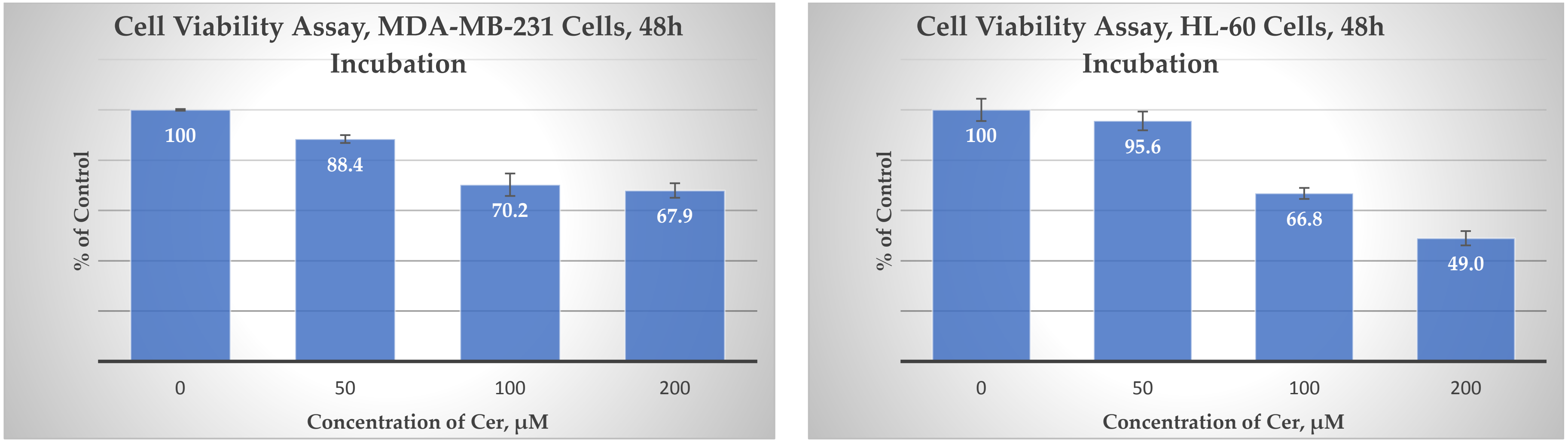

(**a**) (**b**)

Figure S11. Cytotoxicities of total ceramide against (**a**) MDA-MB-231 and (**b**) HL-60 cell lines.

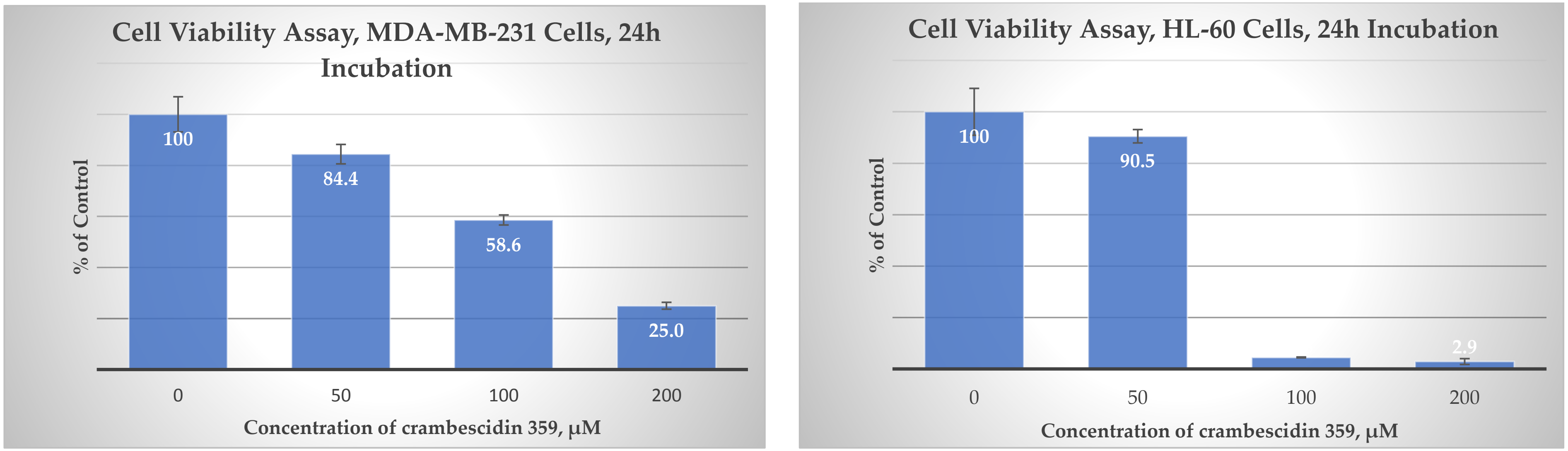

(**a**) (**b**)

Figure S12. Cytotoxicities of crambescidin 359 against (**a**) MDA-MB-231and (**b**) HL-60 cell lines.

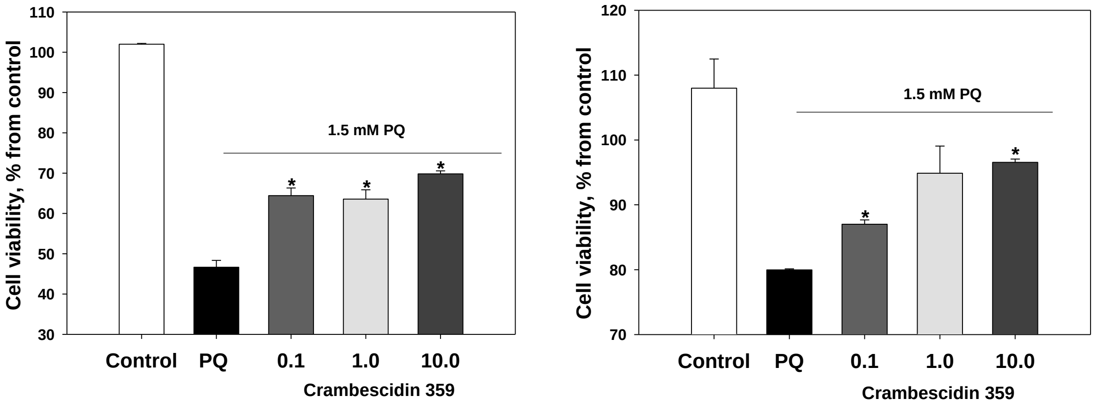

Figure S13. Protective effects of crambescidine 359 against neurotoxicity of paraquat (PQ): (**a**) Neuro-2a cells (24 h pre-incubation with compound **7**) and (**b**) SH-SY5Y cells (48 h pre-incubation with compound **7**). The data are presented as m ± se (n = 3). * $p < 0.05$ compared to cells exposed to paraquat alone.

Crambescidin 359 (**7**) at the highest concentration (100 μM) exhibited a slight neuroprotective effect but triggered intense ROS formation (after 1 h pre-incubation, not shown). The neuroprotective effects of compound **7** were greater for murine (Neuro 2a) than for human (SH-SY5Y) cells. In particular, crambescidin 359 at the concentrations of 0.1, 1.0, and 10.0 μM significantly increased the number of viable murine cells by 38.7±4.1, 36.3±4.9, and 49.7±1.6 %, respectively (Figure S13a). For human cells, crambescidin 359 at the concentration of 10.0 μM increased cell viability by maximum 20.8±0.6% (Figure S13b). Perhaps, compound **7** may inhibit the neurodegenerative activity of paraquat (which is also organic salt containing positively charged nitrogen and anion) by competing with it for binding sites on cells.
